# Influence of the Injection Bias on the Capacitive Sensing of the Test Mass Motion of Satellite Gravity Gradiometers

**DOI:** 10.3390/s24041188

**Published:** 2024-02-11

**Authors:** Hengtong Xu, Jungang Lei, Detian Li, Yunpeng Li, Wenze Tao, Wenyan Zhang, Meng Chen

**Affiliations:** Science and Technology on Vacuum Technology and Physics Laboratory, Lanzhou Institute of Physics, Lanzhou 730000, China

**Keywords:** gradiometers, satellite gravity, capacitive sensing, influence

## Abstract

The performance of the capacitive gap-sensing system plays a critical role in a satellite-based gravity gradiometer that is developed using an electrostatic accelerometer. The capacitive sensing gain mainly depends on the stabilized injection bias amplitude, the gain of the transformer bridge, and the trans-impedance amplifier. Previous studies have indicated that amplitude noise is the main factor influencing the noise of capacitive displacement detection. Analyzing the capacitive gap-sensing system indicates that the amplitude, frequency, phase, and broadband noises of the stabilized injection bias have varying levels of influence on the performance of the detection system. This paper establishes a model to clarify the mentioned effects. The validation of the sub-tests demonstrates that the analysis and evaluation results of various noise coefficients are highly consistent with the model’s predicted outcomes.

## 1. Introduction

With the sequential launches and operations of gravity gradient measurement satellites such as GRACE/COCE, it is vital to develop high precision gravity gradient measurements. The effectiveness of the capacitive gap-sensing system, integral to an onboard gravity gradiometer constructed from an electrostatic accelerometer, plays a pivotal role in achieving the performance of the gradiometer. This system is instrumental in both space gravity experiments and Earth gravity field recovery [1,2,3].

Gravity Field and steady-state Ocean Circulation Explorer (GOCE) was the first satellite mission equipped with a gravitational gradiometer [4,5,6]. Initiated in 1998, the GOCE mission aimed to achieve a noise level of about $5\;mE/\sqrt{Hz}$ in the Electrostatic Gravity Gradiometer (EGG) across a measurement bandwidth (MBW) ranging from 5 mHz to 0.1 Hz [7]. The National Aerospace Research Agency (ONERA) has significantly advanced in developing high-precision detectors capable of measurements with a resolution of up to $10^{-7\;}pF/\sqrt{Hz}$. Additionally, ONERA has examined the sensor output both with and without carrier signals on the capacitance bridge, assessing the impact of stabilized injection bias amplitude on stability [8,9]. Weber et al. developed a capacitive gap-sensing system targeting a resolution near $10^{-6}\;pF/\sqrt{Hz}$, focusing on the 0.1 mHz to 0.1 Hz bandwidth relevant to the Laser Interferometer Space Antenna (LISA). Their findings emphasize the necessity of the relative stability of the stabilized injection bias being better than $2\times 10^{-4}/\sqrt{Hz}$ [10,11]. Based on Armano et al.’s studies, the capacitive detection noise of the orbiting LISA Pathfinder spaceship (LPF) is lower than $1.8\times 10^{-6}\;pF/\sqrt{Hz}$ at frequencies above 1 mHz, whose equivalent displacement detection noise is $2.4\times 10^{-9}\;m/\sqrt{Hz}$. They found that the amplitude of the carrier wave directly influences the displacement detection noise [12]. Dolesi et al. discussed the current design and noise model for the European gravity sensor (GS) in the LISA Technology Package (LTP), featuring a capacitive gap-sensing system with a resolution of $2.4\times 10^{-9}\;m/\sqrt{Hz}$, a 10 µm bias, and a $100\;ppm/\sqrt{Hz}$ stability of the stabilized injection bias [13]. In order to achieve a resolution of $(2\sim 6)\times 10^{-12}\;m/\sqrt{Hz}$ in the frequency band of 5 mHz to 0.1 Hz, the sinusoidal carrier signal employed in the high-precision displacement detection circuit should produce as little residual noise as possible [9,14,15,16,17,18]. In the scope of this research, the stabilized injection bias utilizes a sinusoidal carrier waveform, which aligns fundamentally with the waveform presented in prior scholarly publications. Previous studies evaluated the stabilized injection bias performance using a lock-in amplifier, focusing solely on amplitude noise, but omitting other significant factors.

This paper delves into the principles and noise model of the capacitive gap-sensing system, emphasizing the impact of various factors on the stabilized injection bias. We introduce a model to assess the stabilized injection bias’s influence on the measurement system’s performance, including amplitude, phase, frequency, and broadband noises. This model provides a comprehensive evaluation methodology for assessing the effect of each factor. Finally, we validate the influence of the stabilized injection bias on the displacement detection system through experimental trials, analyzing the contribution of each factor to the total noise under different scenarios.

## 2. Analysis of the Impact of the Carrier Wave on Capacitive Position Sensing

**Figure 1:**
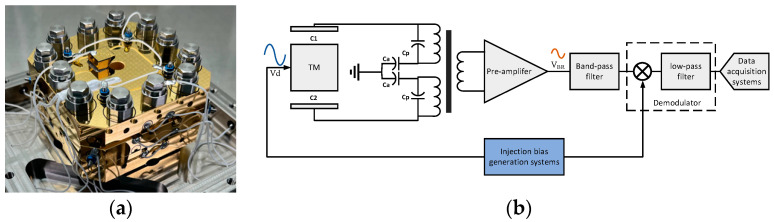
(**a**) Sensor’s internal structure; (**b**) schematic diagram of a high-precision capacitive displacement detection circuit.

Figure 1 illustrates the fundamental principle of the capacitive gap-sensing system in sensors such as accelerometers or gravity gradiometers. The Test Mass (TM), serving as the reference for geodesic motion, must be shielded from external environmental disturbances. Hence, it is encapsulated within an Electrode Housing (EH) and a spacecraft. Multiple channels can be established between TM and EH. For clarity, one sensing channel for the y-ϕ Degree of Freedom (DoF) is shown in Figure 1, while others are omitted for simplicity. The TM and the plates of its EH form capacitances C_1_ and C_2_, respectively. When the TM is centrally positioned, the sensing capacitances C_1_ and C_2_ are equal; otherwise, a differential capacitance ΔC emerges from the capacitors C_1_ and C_2_. The introduction of the stabilized injection bias (*V_d_*) into the sensor, capacitor-transformer bridge, preamplifier, and AC amplifier will modulate the differential capacitance ΔC into an amplitude-modulated signal *V_BR_*.

After demodulating and filtering the amplitude-modulated signal, the data are acquired and archived by the data acquisition system. Our developed system, known as the Stabilized Injection Bias Generation System, adeptly generates *V_d_*. It also concurrently produces a phase-adjustable square wave tailored for demodulation purposes, referred to as the ‘Local Oscillator Signal’. The Stabilized Injection Bias Generation System simultaneously creates *V_d_* and a phase-adjustable square wave for demodulation.

As *V_d_* enters the sensor and displacement detector, the gain yielded by the stabilized injection bias includes the gains of the sensor, preamplifier, AC amplifier, demodulation, and capacitor-transformer bridge-differential transformer bridge. The output of the capacitive gap sensing system can be articulated with Equation (1).

(1) $$u_{o\_s}(t)=K\frac{C_{a}}{C_{a}+C_{p}}\times \frac{2\Delta C}{C_{FB}}\times V_{d}(t)\times \frac{2G_{AMP}}{\pi }$$

where *K* is the transformer coupling coefficient, *C_a_* and *C_p_* are the resonant capacitors in the differential transformer bridge, respectively, *C_FB_* is the feedback capacitor in the pre-amplifier, and *G_AMP_* is the total gain of the AC amplifier and bandpass filter.

Since the injection bias is only a superposition gain without frequency shifting, we set the total gain of the transformer coupling coefficient, the differential transformer bridge, the preamplifier, the AC amplifier, and the bandpass filter in the displacement detection circuit as *G_DIS_*. The *V_BR_* in the displacement detection circuit is only related to the injection bias for fixed values of the differential capacitance Δ*C* and the *G_DIS_* gain. The injection bias is a sine wave with amplitude *V_S_*, a period of *T*_1_, and an angular frequency of $\omega_{1}$. *V_BR_(t)* can be expressed with Equation (2).

(2) $$V_{BR}(t)=G_{DIS}\times V_{d}(t)=G_{DIS}\times V_{S}\cos(\omega_{1}t+\theta )$$

where *θ* is the fixed phase of the sine wave. The local oscillator signal *r(t)* is a square wave of amplitude ±*V_r_* with a period of *T*_2_ and an angular frequency of $\omega_{2}$. The local oscillator signal *r(t)* can be expanded in the following Fourier series.

(3) $$r(t)=\frac{4V_{r}}{\pi }\sum_{n=1}^{\infty }\frac{{\left(-1\right)}^{n+1}}{2n-1}\cos[(2n-1)\omega_{2}t]$$

The demodulation circuit functions can be described in the time domain primarily as a multiplication of *V_BR_(t)* and the local oscillator signal *r(t)*. The result can be expressed as Equation (4).

(4) $$V_{d\mathrm{is}}(t)=G_{DIS}\times V_{d}(t)\times r(t)$$

(5) $$=\frac{2G_{DIS}V_{s}V_{r}}{\pi }\sum_{n=1}^{\infty }\frac{(-1{)}^{n+1}}{2n-1}\cos[\left(2n-1\right)\omega_{2}t-\omega_{1}t-\theta ]+\frac{2G_{DIS}V_{s}V_{r}}{\pi }\sum_{n=1}^{\infty }\frac{(-1{)}^{n+1}}{2n-1}\cos[(2n-1)\omega_{2}t+\omega_{1}t+\theta ]$$

The first and second terms are the differential frequency and sum frequency, respectively. The output of the multiplier passes through a low-pass filter, where the sum of all terms with *n* > 1 and difference frequency terms are filtered out, leaving only *n* = 1 difference frequency terms.

(6) $$V_{dis}=\frac{2G_{DIS}V_{s}V_{r}}{\pi }\cos(\omega_{2}t-\omega_{1}t-\theta )$$

Ideally, the *V_BR_(t)* and oscillator signal have the same frequency, i.e., $\omega_{1}$ = $\omega_{2}=\omega_{0}$. In order to ensure the maximum amplitude and maximum signal-to-noise ratio of the output signal, the phase difference *θ* is usually kept to 0. When using a demodulation circuit with analogue switches, the amplitude of the local oscillator signal can reach 1 V. Therefore, Equation (6) can be simplified to Equation (7), while the output signal is only related to the amplitude of *V_S_*.

(7) $$V_{dis}=\frac{2G_{DIS}V_{s}}{\pi }$$

According to Equations (1) and (7), it is found that although the output signal of the capacitive gap-sensing system undergoes low-frequency fluctuations due to variations in fixed elements such as circuit components, it is more critical to be aware of how the inherent characteristics of the injection bias can also lead to significant low-frequency noise. Consequently, comprehensively understanding the impact of the stabilized injection bias on the capacitive gap-sensing system necessitates a detailed analysis of Equations (6) and (7).

## 3. The Influence of Multiple Factors of the Injection Bias

**Figure 2:**
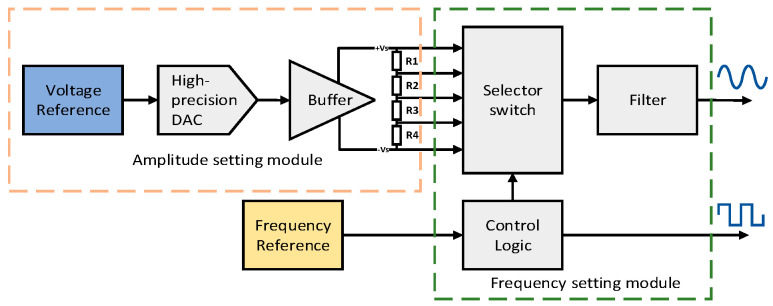
Block diagram of the Stabilized Injection Bias Generation System.

The Stabilized Injection Bias Generation System, as depicted in Figure 2, is a shared resource across all channels. It employs a low-power digital-to-analog converter (DAC) to produce the DC signal, which was used as the signal amplitude. This DC signal is split into four analog levels through a specific timing sequence to create a stepped wave. A fourth-order low-pass filter then smooths this stepped wave into the desired carrier signal. Concurrently, the system generates a square wave signal to facilitate the demodulation process.

However, the components used in the Stabilized Injection Bias Generation System, including voltage and frequency references, are not perfect. These imperfections will inevitably lead to voltage and frequency instabilities, leading to noise in the sine carrier amplitude *V_S_*, the phase difference *θ* between the injection bias and the local oscillator signal, and the injection bias’s angular frequency. Additionally, the system inherently introduces broadband noise superimposed onto the injection bias.

To understand how these bias factors affect the performance of the displacement detection system, it is necessary to adjust Equation (7) to account for these non-ideal conditions. The ideal scenario is outlined in Table 1, where it is assumed that all other factors remain ideal when one factor is varied for simplicity.

### 3.1. Amplitude Noise

The non-idealities of the low-power DAC, such as quantization error, clock jitter, power supply noise, and electromagnetic interference, are significant sources of amplitude noise. Additionally, the 1/*f* noise characteristic of the DAC’s output voltage becomes more pronounced under certain conditions, and the noise increases as the amplitude *V_S_* increases in such cases.

Assuming that an amplitude noise value ${\tilde{V}}_{S}$ is superimposed on the amplitude *V_S_*, the displacement detection output should be consistent with the calculated value of Equation (7) at different values of amplitude *V_S_*. Accordingly, the displacement detection output is

(8) $$V_{dis}=\frac{2}{\pi }(V_{S}+{\tilde{V}}_{S})G_{DIS}$$

**Figure 3:**
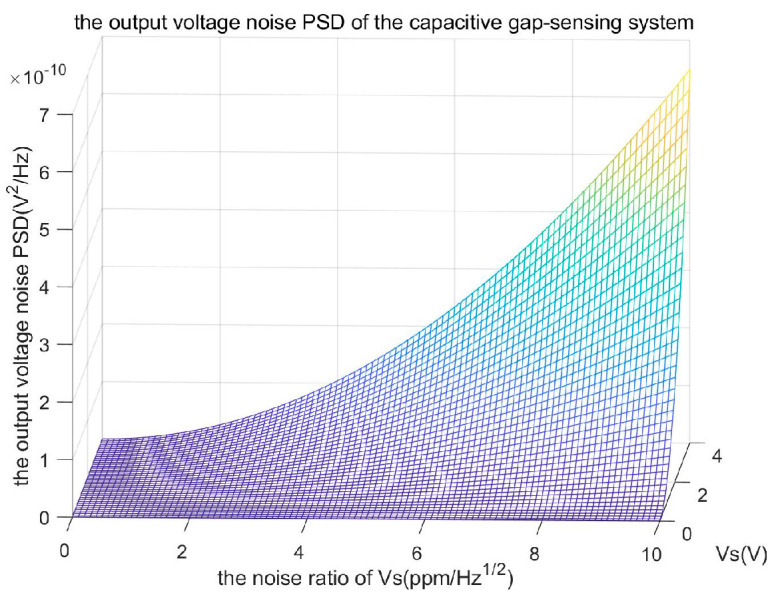
The output voltage noise PSD of the capacitive gap-sensing system.

${\tilde{V}}_{S}$ produces a pair of sidebands on either side of the central frequency of the injection bias in the spectrogram, as the amplitude noise comprises a mixture of noise at several frequencies. Figure 3 shows the simulated results of the capacitive gap-sensing system’s output noise power spectral density (PSD) as the noise ratio of amplitude *V_S_* varies from 0 to $10\;ppm/\sqrt{Hz}$ of the amplitude voltage.

The output voltage noise PSD of the capacitive gap-sensing system reaches $6.48\times 10^{-12}\;V^{2}$/Hz, $1.62\times 10^{-10}\;V^{2}$/Hz, and $6.48\times 10^{-10}\;V^{2}$/Hz, respectively, when the amplitude changes from 0 V to 4 V, and the noise ratio of amplitude *V_S_* is $1\;ppm/{Hz}^{0.5}$, $5\;ppm/\sqrt{Hz}$, and $10\;ppm/\sqrt{Hz}$. As the amplitude noise factor increases, the displacement noise power increases as a quadratic term. Therefore, the amplitude noise factor should be minimized to reduce the total noise voltage.

### 3.2. Frequency Noise and Phase Noise

**Figure 4:**
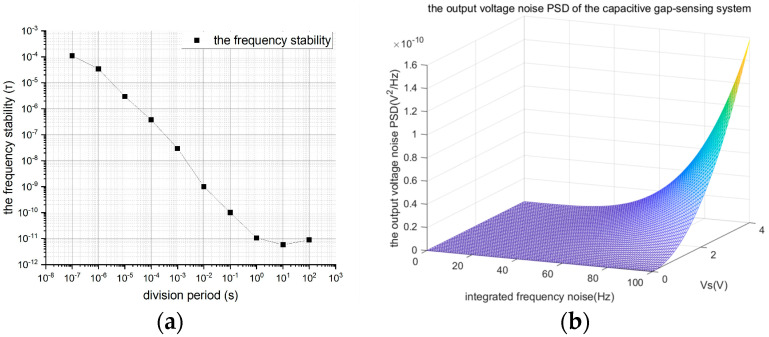
(**a**) The frequency stability curve of a typical 10 MHz crystal oscillator (8607-BE) and (**b**) the output voltage noise’s PSD of the displacement detection circuit.

The crystal oscillator frequency in the system provides the main frequency reference of the Stabilized Injection Bias Generation System. Figure 4a displays the frequency stability curve of a typical 10 MHz crystal oscillator (8607-BE). It is evident from this curve that the stability of the crystal frequency is positively correlated with the sampling time (division period), meaning that the longer the sampling time, the higher the stability of the crystal frequency. Notably, the frequency stability improves by an order of magnitude with each tenfold increase in the crossover period—a hallmark trait of high-stability crystal oscillators. Concurrently, the response time of switching devices and the parasitic effects within the system’s components can diminish the frequency stability. Consequently, all these factors commonly lead to the production of the pronounced frequency and phase noises in the interplay between the injection bias and the local oscillator signal.

The frequency difference ∆ω between the injection bias and demodulation frequencies can be regarded as frequency modulation (FM). The injection bias can be expressed as follows

(9) $$e(t)=V_{S}\cos(\omega_{1}t+\beta \sin\omega_{m}t)$$

where β is the maximum frequency difference, and $\omega_{m}$ is the modulation frequency of the frequency difference. When the modulation index is very small (narrowband FM), i.e., *β* << π/2. Let *V_S_* be 1 V, we have

(10) $$\cos(\beta \sin\omega_{m}t)\approx 1$$

(11) $$\sin(\beta \sin\omega_{m}t)\approx \beta \sin\omega_{m}t$$

Therefore,

(12) $$e(t)=\cos\omega_{1}t+\frac{\beta }{2}\cos(\omega_{1}+\omega_{m})t-\frac{\beta }{2}\cos(\omega_{1}-\omega_{m})t$$

Equation (13) demonstrates that the spectrum of a narrowband FM wave consists of a central frequency and two side frequency components. The results obtained in the lock-in amplifier circuit can be easily confused because the narrowband FM and AM spectra are comparable.

The phase noise *θ* at the injection bias can be considered as phase modulation (PM). For single-tone phase modulation, the injection bias can be expressed as follows

(13) $$e(t)=V_{S}\cos(\omega_{1}t+\theta_{d}\sin\omega_{m}t)$$

where $\vartheta_{d}$ rad is the maximum phase shift. The PM spectrum with phase disturbance $\vartheta_{d}$ rad is identical to the FM spectrum with frequency disturbance $\vartheta_{d}$ rad. Therefore, PM and FM modulations can be transformed into each other according to the following relationship.

(14) $$\theta_{d}=\frac{\Delta f_{peak}}{f_{m}}\times 2\pi$$

where $f_{m}$ is the main frequency of the injection bias. After modulating the phase and frequency noises, the displacement output can be expressed with Equation (15).

(15) $$u_{dis}(t)=\frac{2G_{DIS}V_{S}}{\pi }\cos(\Delta \theta )$$

Thus, the angular frequency difference ∆*ω* and the phase difference ∆ϑ can be combined to form the integrated frequency noise $f_{int}$. Figure 4b shows the simulated results of the output noise PSD of the capacitive gap-sensing system as the integrated frequency noise $f_{int}$ varies from 0 to 100 Hz.

When the amplitude varies from 0 V to 4 V, the maximum phase (frequency) variations of 10 Hz, 50 Hz, 80 Hz, and 100 Hz will contribute to the noise power of $1.57\times 10^{-10}\;V^{2}$/Hz, $\;1.03\times 10^{-9}\;V^{2}$/Hz, and$\;2.52\times 10^{-9}\;V^{2}$/Hz, respectively. According to Equation (6), the displacement noise power increases significantly as the phase (frequency) noise increases. Therefore, phase (frequency) noise should be minimized to reduce the total noise voltage. More seriously, the combined frequency noise $f_{int}$ in the Stabilized Injection Bias Generation System positively correlates with the amplitude *V_S_*. Reducing the main frequency may optimize both the frequency and phase noises, while it can only be adjusted appropriately considering the performance of the capacitive gap-sensing system.

### 3.3. Broadband Noise

The capacitive gap-sensing system can process signals in the frequency range of (*f_0_* − *f_LPF_*, *f*_0_ + *f_LPF_*) Hz, where *f_LPF_* is the low-pass filter’s cutoff frequency in the demodulation circuit. Due to its device noise, temperature noise, and other noises, a broadband noise *n(t)* is inevitably superimposed on the output of the Stabilized Injection Bias Generation System. Therefore, when the noise *n(t)* is superimposed on the (*f*_0_ − *f_LPF_*, *f*_0_ + *f_LPF_*) Hz band, *n(t)* will be demodulated to the (0, 0 + *f_LPF_*) Hz band. Based on the above analysis, the displacement detection output signal is represented with Equation (16).

(16) $$u_{dis}(t)=\frac{2G_{DIS}V_{S}}{\pi }+n(t)$$

### 3.4. Total Noise

In the non-ideal case, the equivalent displacement output introduces noise $\tilde{V_{S}}$, which depends on the amplitude *V_S_* of the carrier, the phase difference ∆θ, and the angular frequency difference ∆ω between the carrier and the local oscillator signal. Thus, Equation (6) can be expanded to Equation (17).

(17) $$u_{dis}(t)=\frac{2G_{DIS}[V_{S}+\tilde{V_{S}}(t)]}{\pi }\cos[\Delta \omega (t)t+\Delta \theta (t)]+n(t)$$

## 4. Experimental Results

### 4.1. Amplitude Noise

In the previous section, Figure 2 shows the block diagram of the Stabilized Injection Bias Generation System. The amplitude is set by the DAC, which can be measured directly. A high-precision ADC (LTC2508) is employed in the time domain to acquire the voltage’s amplitude. The LTC2508-based acquisition circuit’s data processing parameters are shown in Table 2. The amplitude noise’s PSD is shown in Table 3 when the amplitude varies from 1 V to 4 V. This paper employs a Hanning-window for the one-sided spectrum to perform the PSD processing based on the Fourier transform.

As the amplitude increases, the amplitude noise’s PSD increases accordingly, consistent with the theoretical analysis. At 100 mHz, the maximum noise ratio of amplitude *V_S_* is $1.29\;ppm/\sqrt{Hz}$; at 5 mHz, the maximum noise ratio of amplitude *V_S_* is $2.49\;ppm/\sqrt{Hz}$ due to 1/*f* noise.

### 4.2. Frequency Noise and Phase Noise

**Figure 5:**
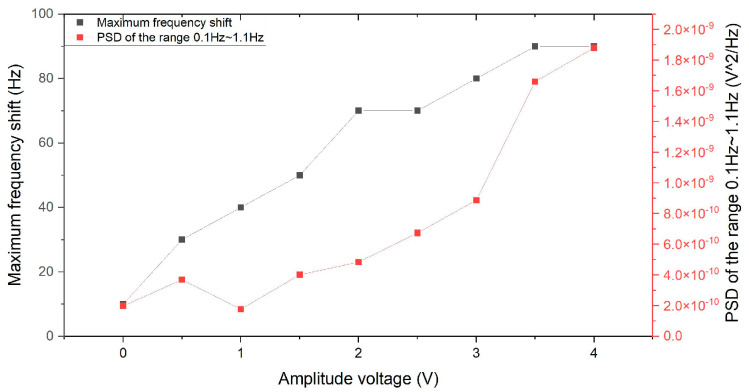
The combined frequency noise $f_{int}$ versus the amplitude voltage value.

Here, we evaluate the combined frequency noise $f_{int}$ using an Agilent E4440A spectrum analyzer, where its parameters are shown in Table 4. The combined frequency noise combines phase and frequency noises using the main frequency. In the RBW range, the combined frequency noise $f_{int}$ versus the amplitude voltage value is shown in Figure 5. The combined frequency noise $f_{int}$ is positively related to the amplitude. By linear fitting, for every 1 V increase in amplitude, the combined frequency noise is correspondingly increased by 19.90 ± 1.57 Hz.

### 4.3. Broadband Noise and Total Noise

**Figure 6:**
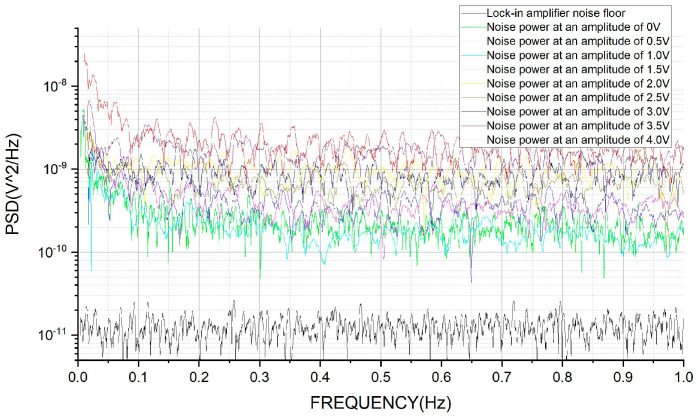
The background noise of the lock-in amplifier and the noise when setting the stabilized injection bias amplitude to 0 to 4 V.

Here, the total noise is evaluated by a lock-in amplifier, which will demodulate the stabilized injection bias with different amplitudes. The noise’s PSD of the obtained signals by the lock-in amplifier can evaluate the performance of the stabilized injection bias, as shown in Figure 6. Figure 6 includes the background noise power spectrum of the lock-in amplifier, which is $3.3\times 10^{-6}\;V/\sqrt{Hz}$ (1.089 × 10^−11^ V^2^/Hz) over the range of 5 mHz to 100 mHz.

When the frequency tends to 0 Hz, the 1/*f* noise plays a vital role in elevating the noise significantly. Moreover, 1/*f* noise, or flicker noise, can also originate internally, particularly from components such as operational amplifiers. The equivalent input noise voltage and current of operational amplifiers are significant sources of 1/*f* noise, especially in low-frequency applications. This internal source of noise is often related to defects and imperfections in the semiconductor material and varies with the operating conditions of the amplifier. To analyze the noise powers in the unit frequency more accurately, the PSDs in the range of 5 mHz to 1.005 Hz and 0.1 Hz to 1.1 Hz are selected to obtain the average PSD values, as shown in Table 5.

### 4.4. Noise Analysis

To corroborate the theoretical predictions with empirical data, we synthesized the amplitude noise, phase noise, frequency noise, and broadband noise at frequencies of 5 mHz and 0.1 Hz using Equation (17). The PSD of the synthetic noise, as modeled, is also presented in Table 5.

**Figure 7:**
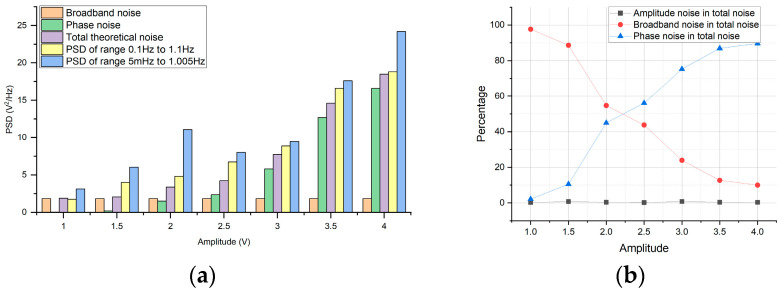
(**a**) Total noise power versus amplitude, broadband, and frequency (phase) noises. (**b**) The proportion of each noise.

The synthetic noise’s PSD by model and all factors are represented in Figure 7. Since a low-noise voltage generation circuit is employed to generate the amplitude of the stabilized injection bias, the PSD of the amplitude noise is two orders of magnitude lower than that of the total noise. The superposition of broadband and phase noises has reached over 90% of the total noise power. When *V_S_* < 2.25 V, the broadband noise accounts for more than 50% of the total noise. As the amplitude increases, the proportion of phase frequency noise power in the total noise power gradually increases.

As demonstrated in Figure 7, the test results align with the theoretical analysis using Equation (17), demonstrating that the PSD synthesized by the model is consistent with the average PSD obtained between 0.1 Hz and 1.1 Hz. However, notable fluctuations occur in the average PSD within the low-frequency range, primarily due to the influence of external factors on 1/f noise.

While the quantification of 1/*f* noise remains somewhat elusive, the analysis of carrier performance in this study aligns closely with the noise measurements exhibited in the higher frequency band (0.1 Hz to 1.1 Hz). In addition, this study aims to assess the impact of the stabilized injection bias on the performance of the measurement system and the effectiveness of the evaluation method. To this end, we have utilized the model to evaluate and enhance the circuit topology, component selection, and control timing within the Stabilized Injection Bias Generation System. The noise’s PSD value of the stabilized injection bias is shown in Table 5. Finally, the stabilized injection bias’s stability is <$17.4\;ppm/\sqrt{Hz}$ at 100 mHz and <$55.5\;ppm/\sqrt{Hz}$ at 5 mHz, indicating that a good stability was achieved in the present work.

## 5. Conclusions

The performance of the stabilized injection bias has a significant impact on the performance of the capacitive gap-sensing system, which has been systematically studied and analyzed at present. First, in this work, we provide an in-depth theoretical decomposition of the performance of stabilized injection bias and present a detailed model to verify the effect of the stabilized injection bias on the measurement system’s performance. This model indicates that the stabilized injection bias’s amplitude, frequency, phase, and broadband noises can significantly increase the noise of the capacitive gap-sensing system. At the same time, to comprehensively assess the individual impacts of the four noise factors on the detection accuracy of the capacitive gap-sensing system, we have specifically developed a series of different evaluation methods to conduct an in-depth analysis and assessment of each noise factor separately. Finally, this evaluation method is employed to investigate and optimize a self-developed injection bias generation system. The synthetic noise PSD by the developed model is compatible with the measured value. In addition, the experiment results indicate that the broadband and frequency (phase) noises accounted for over 90% of the total noise. Broadband noise accounts for over 50% of the noise at low amplitudes. We ultimately achieve a sine wave stability of <$17.4\;ppm/\sqrt{Hz}$ at 100 mHz and <$55.5\;ppm/\sqrt{Hz}$ at 5 mHz through improvements to the self-developed injection bias generation system.

## Figures and Tables

**Table 1:** Typical parameters in the carrier wave analysis.

The Injection Bias Parameters	Unit	Typical Value
Amplitude value *V_S_*	V	0~4
Phase difference *θ*	rad	0
Angular frequency difference ∆ω	rad	0
Frequency *f*0	kHz	100.0
Angular frequency $\omega_{0}$	rad	$2\pi \times 1\times 10^{5}$
Noise bandwidth (NBW)	Hz	1
Equivalent gain A	dB	0

**Table 2:** LTC2508-based acquisition circuit and data processing parameters.

Parameters	Unit	Typical Value
Sampling frequency	MHz	1.024
Anti-aliasing frequency	Hz	2
Window functions	-	Hanning

**Table 3:** Noise’s PSD for each amplitude.

Amplitude			1 V	1.5 V	2 V	2.5 V	3 V	3.5 V	4 V
Amplitude noise spectral density	5 mHz	$10^{-10}\;V^{2}/Hz$	0.04	0.14	0.24	0.37	0.43	0.34	0.49
	100 mHz		0.01	0.04	0.03	0.02	0.15	0.14	0.14
Amplitude noise factor	5 mHz	ppm/√Hz	2.00	2.49	2.45	2.43	2.19	1.67	1.75
	100 mHz		1.33	0.87	0.57	1.29	1.07	0.94	1.00

**Table 4:** Agilent E4440A spectrum analyzer parameters.

The Carrier Wave Parameters	Unit	Typical Value
Resolution bandwidth (RBW)	Hz	1.8
The conversion factor of the resolution bandwidth to noise bandwidth kn	-	1.128

**Table 5:** The average PSD and synthetic noise PSD by model and the stabilized injection bias stability.

Amplitude	V	1	1.5	2	2.5	3	3.5	4
The average PSD in the range of 5 mHz~1.005 Hz	$10^{-10}\;V^{2}/Hz$	3.14	6.05	11.1	8.02	9.48	17.6	24.2
The average PSD in the range of 0.1 Hz~1.1 Hz	$10^{-10}\;V^{2}/Hz$	1.77	4.01	4.84	6.74	8.87	16.6	18.8
Synthetic noise’s PSD by model@ 5 mHz	$10^{-10}\;V^{2}/Hz$	1.91	2.13	3.47	4.37	7.84	14.68	18.63
Synthetic noise’s PSD by model@ 0.1 Hz	$10^{-10}\;V^{2}/Hz$	1.89	2.09	3.38	4.23	7.73	14.60	18.49
The stabilized injection bias stability@ 5 mHz	$ppm/\sqrt{Hz}$	55.5	38.1	34.1	32.8	21.3	20.5	31.8
The stabilized injection bias stability@ 0.1 Hz	$ppm/\sqrt{Hz}$	14.4	16.5	17.4	10.5	7.5	12.5	10.8

## Data Availability

The data used to support the findings of this study are available upon request from the corresponding author.

## References

[1] Drinkwater M., Kern M. (2006). Calibration and Validation Plan for L1b Data Products.

[2] Drinkwater M., Floberghagen R., Haagmans R., Muzi D., Popescu A., Beutler G.B., Drinkwater M., Rummel R., von Steiger R. (2003). GOCE: ESA’s First Earth Explorer Core Mission, Earth Gravity Field from Space-from Sensors to Earth Sciences.

[3] Sünkel H. (1986). Mathematical and Numerical Techniques in Physical Geodesy. Lecture Notes in Earth Sciences.

[4] Rummel R., Yi W., Stummer C. (2011). GOCE gravitational gradiometry. J. Geod..

[5] Marque J., Christophe B., Liorzou F., Bodovillé G., Foulon B., Guérard J., Lebat V. The ultra sensitive accelerometers of the ESA GOCE mission. Proceedings of the 59th International Astronautical Congress (IAC-08-B1. 3.7).

[6] Bergé J., Christophe B., Foulon B. GOCE accelerometers data revisited: Stability and detector noise. Proceedings of the ESA Living Planet Symposium.

[7] Johannessen J.A., Balmino G., Le Provost C., Rummel R., Sabadini R., Sünkel H., Tscherning C., Visser P., Woodworth P., Hughes C. (2003). The European gravity field and steady-state ocean circulation explorer satellite mission its impact on geophysics. Surv. Geophys..

[8] Josselin V., Touboul P., Kielbasa R. (1999). Capacitive detection scheme for space accelerometers applications. Sens. Actuators A Phys..

[9] Touboul P., Foulon B., Willemenot E. (1999). Electrostatic space accelerometers for present and future missions. Acta Astronaut..

[10] Weber W.J., Cavalleri A., Dolesi R., Fontana G., Hueller M., Vitale S. (2002). Position sensors for LISA drag-free control. Class. Quantum Gravity.

[11] Weber W.J., Bortoluzzi D., Cavalleri A., Carbone L., Da Lio M., Dolesi R., Fontana G., Hoyle C.D., Hueller M., Vitale S. (2003). Position sensors for flight testing of LISA drag-free control. Gravitational-Wave Detection.

[12] Armano M., Audley H., Auger G., Baird J., Bassan M., Binetruy P., Born M., Bortoluzzi D., Brandt N., Caleno M. (2017). Capacitive sensing of test mass motion with nanometer precision over millimeter-wide sensing gaps for space-borne gravitational reference sensors. Phys. Rev. D.

[13] Dolesi R., Bortoluzzi D., Bosetti P., Carbone L., Cavalleri A., Cristofolini I., DaLio M., Fontana G., Fontanari V., Foulon B. (2003). Gravitational sensor for LISA and its technology demonstration mission. Class. Quantum Gravity.

[14] Christophe B., Marque J., Foulon B. In-orbit data verification of the accelerometers of the ESA GOCE mission. Proceedings of the Annual Meeting of the French Society of Astronomy and Astrophysics, SF2A-2010.

[15] Gan L., Mance D., Zweifel P. (2011). Actuation to sensing crosstalk investigation in the inertial sensor front-end electronics of the laser interferometer space antenna pathfinder satellite. Sens. Actuators A Phys..

[16] Li K., Bai Y., Hu M., Qu S., Wang C., Zhou Z. (2020). Amplitude stability analysis and experimental investigation of an ac excitation signal for capacitive sensors. Sens. Actuators A Phys..

[17] Li K., Chen D., Li D., Wang C., Yang X., Hu M., Bai Y., Qu S., Zhou Z. (2023). A novel modem model insensitive to the effect of the modulated carrier and the demodulated-signal phase adapted for capacitive sensors. Measurement.

[18] Mance D., Zweifel P., Ferraioli L., ten Pierick J., Meshksar N., Giardini D., LISA Pathfinder Collaboration (2017). GRS electronics for a space-borne gravitational wave observatory. Journal of Physics: Conference Series.

